# Oxidative balance score is associated with the risk of diabetic kidney disease in patients with type 2 diabetes mellitus: evidence from NHANES 2007–2018

**DOI:** 10.3389/fnut.2024.1499044

**Published:** 2024-12-19

**Authors:** Yu Liang, Zhonggao Xu, Wanning Wang

**Affiliations:** Department of Nephrology, The First Hospital of Jilin University, Changchun, Jilin, China

**Keywords:** diabetic kidney disease, oxidative balance score, NHANES, diabetes mellitus, oxidative stress

## Abstract

**Objective:**

The oxidative balance score (OBS) is a comprehensive measure of oxidative stress that is calculated from the combined prooxidant and antioxidant scores of 16 dietary components and four lifestyle factors. This study aimed to evaluate the relationship between OBS and the risk of diabetic kidney disease (DKD) in individuals with Type 2 diabetes mellitus (T2DM).

**Methods:**

Data were obtained from the NHANES. A cross-sectional study was conducted using multiple logistic regression. Covariate effects of this relationship were also examined using subgroup analysis.

**Results:**

We evaluated 3,669 T2DM participants, among whom DKD prevalence was 30.87%. In a fully adjusted logistic regression model, the risk of DKD among participants with OBS, lifestyle OBS, and dietary OBS in the highest quartile group was 0.50 times (95% CI: 0.39 to 0.65), 0.54 times (95% CI: 0.41–0.71), and 0.45 times (95% CI: 0.32–0.63), respectively, than that in the lowest quartile group, respectively. In addition, participants who scored in the top quartiles of OBS were more likely to possess higher levels of education and income. A stratified analysis demonstrated the robustness of these findings.

**Conclusion:**

OBS negatively correlates with the risk of DKD among individuals with T2DM.

## 1 Introduction

Diabetic kidney disease (DKD) is a common and serious complication of type 2 diabetes mellitus (T2DM), and is characterized by proteinuria and a decreased glomerular filtration rate. As the leading cause of chronic kidney disease (CKD) and end-stage renal disease (ESRD) worldwide (1–3), DKD significantly impairs patients’ quality of life and increases the risk of cardiovascular events and premature death. CKD is predicted to become the fifth most common cause of death globally by 2040 (4). With an estimated 642 million individuals projected to have T2DM globally with approximately 30–40% of them potentially developing DKD (5, 6), there is an urgent need for effective strategies to assess and manage DKD risk (7).

Oxidative stress (OS), resulting from an imbalance between pro-oxidants and antioxidants (8), plays a pivotal role in the pathogenesis of DKD by promoting excessive production of reactive oxygen species (ROS) and inducing inflammation in kidney tissues (9–12). OS is linked to various metabolic factors related to diabetes (13), and modifiable exogenous factors such as diet and lifestyle are crucial for maintaining oxidative balance. The oxidative balance score (OBS) is a composite measure that reflects the overall balance of antioxidants and pro-oxidants consumed through diet and lifestyle factors (14–18), including nutrient intake, smoking, alcohol consumption, physical activity, and body mass index (BMI). Pro-oxidant factors such as smoking, alcohol consumption, iron intake, and high-fat diets can increase ROS levels (19–22), whereas antioxidants such as vitamin C and carotenoids help mitigate oxidative damage (23, 24). While epidemiological studies have assessed the association between OBS and various diseases—including periodontitis, non-alcoholic fatty liver disease, and others (25–27)—its relationship with DKD in patients with T2DM remains underexplored.

Previous research has shown that a high OBS is associated with better glycemic control (28) and a reduced risk of diabetes, and that this association is influenced by sex (29), blood pressure (30), etc. Additionally, a close relationship exists between high overall dietary quality and low prevalence of T2DM among middle-aged and elderly individuals (31). Although some cohort studies have examined the impact of dietary quality on CKD development (32–34), none have specifically investigated how OBS affects DKD in T2DM patients. Therefore, in this cross-sectional study utilizing data from the National Health and Nutrition Examination Survey (NHANES) 2007–2018, we investigated the association between OBS and the risk of DKD among individuals with T2DM. By calculating OBS based on 16 dietary components and four lifestyle factors, we evaluated the relationship using multiple logistic regression models and conducted subgroup analyses to assess the consistency of our findings. The elucidation of the association between OBS and DKD should help provide a theoretical foundation for early clinical identification of DKD.

## 2 Materials and methods

### 2.1 Study sample

Data were obtained from the National Health and Nutrition Examination Survey (NHANES), a nationally representative, multi-stage research initiative administered by the National Center for Health Statistics that evaluates the health and diet of Americans. This research program involves the collection of data using a combination of laboratory and physical examinations and the administration of questionnaires in various populations. Informed consent was provided by all participants in writing. Comprehensive datasets are available via the NHANES platform, hosted on https://www.cdc.gov/nchs/nhanes/.

**Figure 1:**
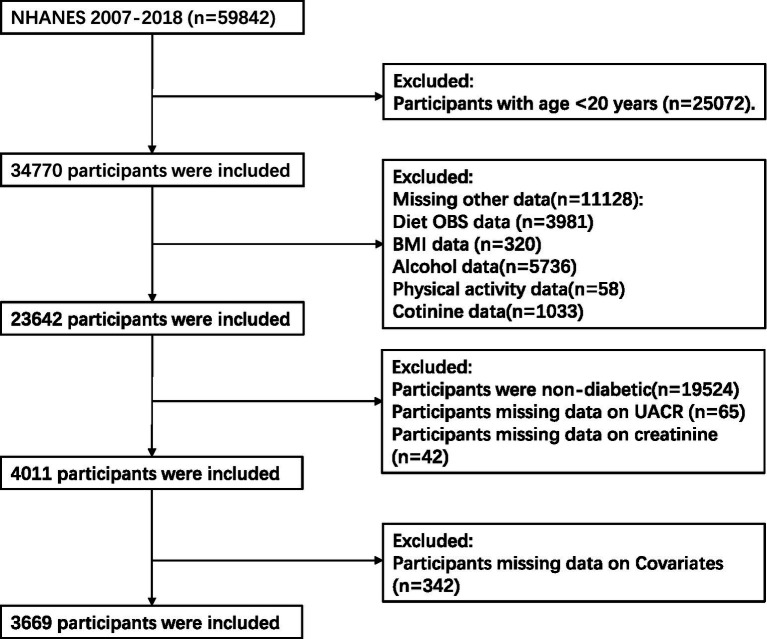
Flowchart describing the study sample. NHANES, National Health and Nutrition Examination Survey; OBS, oxidative balance score; BMI, body mass index; UACR, urinary albumin/creatinine ratio.

Our study included 59,842 individuals who participated in six consecutive 2-year NHANES survey cycles performed during 2007 to 2018 were considered for inclusion in the present study. The exclusion criteria comprised: (1) age < 20 years (*n* = 25,072); (2) incomplete data regarding dietary and lifestyle OBS components (3,981 participants for whom dietary OBS information was missing, 320 for whom no body mass index (BMI) data were available, 5,736 for whom alcohol consumption data were missing, 58 for whom physical activity information was missing, and 1,033 for whom no cotinine data were available); (3) the absence of T2DM (*n* = 19,524); (4) missing urinary albumin/creatinine ratio (*n* = 65) or creatinine (*n* = 42) data; and (5) missing information regarding covariates (*n* = 342). After the application of these criteria, data from 3,669 participants remained for analysis (Figure 1).

### 2.2 Exposures

The OBS was calculated using the method used in previous studies, using pro-oxidant and antioxidant data derived from 16 dietary components and four other lifestyle factors (35). The 16 dietary factors were fiber, *β*-carotene, riboflavin, niacin, vitamin B6, total folate, vitamin B12, vitamin C, vitamin E, calcium, magnesium, zinc, copper, selenium, total fat, and iron, which were derived from the mean intakes of nutrients identified using 24-h dietary recall interviews. Nutrients obtained from supplements, antacids, and medication were not included. Two dietary recall interviews were conducted, one that was conducted face-to-face at the Mobile Examination Center, and another that was telephonically administered within a window of 3 to 10 days thereafter. The four lifestyle factors were physical activity, BMI, alcohol consumption, and smoking habits. Of these 20 components, total fat, iron, alcohol, BMI, and smoking are classified as pro-oxidants, while the rest are classified as antioxidants. Smoking habits were assessed through the measurement of serum cotinine concentration.

Alcohol consumption was categorized according to the amount consumed, with heavy drinkers (≥15 g/d for females and ≥ 30 g/d for males), non-heavy drinkers (0–15 g/d for females and 0–30 g/d for males), and non-drinkers assigned scores of 0, 1, and 2, respectively (36). Physical activity was quantified using metabolic equivalents (METs) as low (<400 MET minutes per week), moderate (400–1,000 MET minutes per week), or high (>1,000 MET minutes per week) (37), with 0, 1 and 2 points being awarded, respectively.

With the exception of physical activity, the participants were allocated to sex-specific tertiles according to each variable. The score of each element was added to calculate the total OBS. Antioxidant exposure was scored as 0 to 2 points, for tertiles 1 to 3 respectively, and pro-oxidant exposure was scored in the reciprocal fashion. Thus, a high OBS score indicated more substantial antioxidant exposure (Table 1).

**Table 1:** Oxidative balance score allocation scheme.

OBS components	Male			Female		
	0	1	2	0	1	2
Dietary OBS components						
Dietary fiber (g/d)	<12.5	12.5–19.85	≥19.85	<10.6	10.6–16.45	≥16.45
Carotene (RE/d)	<48.83	48.83–161.25	≥161.25	<50.04167	50.04167–170.875	≥170.875
Riboflavin (mg/d)	<1.622	1.622–2.325	≥2.325	<1.2945	1.2945–1.843	≥1.843
Niacin (mg/d)	<20.263	20.263–29.0615	≥29.0615	<15.3565	15.3565–21.712	≥21.712
Vitamin B6 (mg/d)	<1.542	1.542–2.3185	≥2.3185	<1.2005	1.2005–1.75	≥1.75
Vitamin B12 (mcg/d)	<3.205	3.205–5.53	≥5.53	<2.445	2.445–4.165	≥4.165
Vitamin C (mg/d)	<37.25	37.25–86	≥86	<36.1	36.1–84.25	≥84.25
Vitamin E (ATE) (mg/d)	<5.54	5.54–9.05	≥9.05	<4.735	4.735–7.435	≥7.435
Calcium (mg/d)	<659.5	659.5–1,023	≥1,023	<563	563–856	≥856
Magnesium (mg/d)	<237.5	237.5–337	≥337	<194	194–271	≥271
Zinc (mg/d)	<8.89	8.89–13.2	≥13.2	<6.63	6.63–9.78	≥9.78
Copper (mg/d)	<0.961	0.961–1.376	≥1.376	<0.806	0.806–1.128	≥1.128
Selenium (mcg/d)	<94.55	94.55–62.955	≥62.955	<71.65	71.65–101.5	≥101.5
Iron (mg/d)	≥17.01	11.645–17.01	<11.645	≥13.46	9.23–13.46	<9.23
Total fat (g/d)	≥95.27	63.03–95.27	<63.03	≥74.005	48.755–74.005	<48.755
Total folate (mcg/d)	<289	289–451	≥451	<240	240–353.5	≥353.5
Lifestyle OBS components						
Physical activity (MET-minute/week)	<400	400–1,000	>1,000	<400	400–1,000	>1,000
Alcohol (g/d)	≥ 30	0–30	non-drinkers	≥15	0–15	non-drinkers
Body mass index (kg/m^2^)	≥33.4	28.27–33.4	<28.27	≥36.6	30.25–36.6	<30.25
Cotinine (ng/mL)	≥0.259	0.019–0.259	<0.019	≥0.118	0.016–0.118	<0.016

OBS, oxidative balance score; RE, retinol equivalent; ATE, alpha-tocopherol equivalent; MET, metabolic equivalent.

### 2.3 Outcome

The criteria used for the diagnosis of T2DM were as follows: a previous diagnosis of diabetes made by a doctor or healthcare professional, a fasting glucose concentration ≥ 7.0 mmol/L, a glycosylated hemoglobin (HbA1c) level of 6.5% or higher, or the contemporaneous administration of insulin or antidiabetic medication. Individuals with T2DM met the criteria for a diagnosis of DKD if their urinary albumin/creatinine ratio (UACR) was ≥30 mg/g and/or their estimated glomerular filtration ratio (eGFR) was <60 mL/min/1.73 m^2^ (38). eGFR was determined using the CKD Epidemiology Collaboration (CKD-EPI) equation (39).

### 2.4 Covariates

A literature review and clinical observations informed the inclusion of covariates that could influence the relationship between OBS and DKD (40–42) (refer to Supplementary Tables S2–S4). These covariates included sex, education level, race, poverty-to-income ratio (PIR), hypertension, and hyperlipidemia. High blood pressure (HBP) was defined based on a healthcare professional’s diagnosis, the contemporaneous administration of medication for hypertension, a mean systolic blood pressure (SBP) ≥140 mmHg, and/or a mean diastolic blood pressure (DBP) ≥90 mmHg. Hyperlipidemia was defined based on a self-reported previous diagnosis of hyperlipidemia by a physician or healthcare professional or abnormal circulating lipid concentrations (total cholesterol ≥6.216 mmol/L, low-density lipoprotein-cholesterol ≥4.144 mmol/L, triglycerides ≥2.26 mmol/L, and/or HDL-cholesterol <1.036 mmol/L).

### 2.5 Statistical analysis

The baseline characteristics of the participants, stratified according to OBS quartile, were studied. To calculate the combined sample weights for participants included in the present analysis, NHANES Analytic Guidelines were followed. In particular, the 2-year dietary subsample weights were divided by 6 (the number of cycles included from 2007 to 2018) to create 12-year weights. For continuous data, the study-weighted mean (95% CI) and *p*-value obtained using study-weighted linear regression are quoted. The categorical variables are reported using a survey-weighted percentage (95% CI) and *p*-value obtained using study-weighted Chi-square test. To measure the impact of missing data on the results, we included and excluded missing values for sensitivity analysis to determine whether the results were consistent. After adjustment for potential confounders, the relationship between the risk of DKD among patients with diabetes and OBS was analyzed using three multivariate logistic regression models. The application of the variance inflation factor in the adjusted models precluded any possibility of collinearity. Model 1 was unadjusted; Model 2 was adjusted for sex, age, and ethnicity; and Model 3 was adjusted for these variables plus educational level, PIR, hypertension, and hyperlipidemia. To further examine the robustness of the findings, a stratified analysis was performed to explore the coherence of the results among distinct subgroups. Furthermore, a restricted cubic spline model with three knots was used in order to more accurately determine whether there is a nonlinear dose–response relationship between OBS and DKD.

Analysis was conducted with R packages (R Foundation for Statistical Computing, Vienna, Austria, version 4.2.0) and Empowerment software.^1^ *p* < 0.05 was regarded as indicating statistical significance.

## 3 Results

### 3.1 Baseline characteristics of the participants

We analyzed data from 3,669 eligible participants that represented a weighted US population of 121,497,866, with men accounting for 56.69% of the number. Of these, 30.87% had been diagnosed with DKD. The participants were allocated to groups according to the quartile of OBS as follows: Q1, Q2, Q3, and Q4 included 865, 904, 977, and 923 participants, respectively. Those in Group Q1 had 4–13 points, those in Group Q2 had 14–19 points, those in Group Q3 had 20–25 points, and those in Group Q4 had 26–36 points. As the quartile number increased, the risk of the participants with DKD decreased. The characteristics of the participants, grouped into quartiles based on their OBS, are shown in Table 2. Among the OBS quartiles, race, educational level, and PIR significantly differed (all *p* < 0.001). The majority of the participants were non-Hispanic white and male. Individuals in the top quartile group of OBS had a higher level of educational attainment and higher PIR than those in the bottom quartile.

**Table 2:** Baseline data of the participants, categorized according to OBS quartile.

	Total	Q1 (4–13)	Q2 (14–18)	Q3 (19–24)	Q4 (25–35)	*P*-value
Sample number	3,669	865	904	977	923	
Weighted number	121,497,866	23,720,928	27,317,317	32,885,551	37,574,070	
Age (years)	58.40 (57.78, 59.02)	59.04 (57.47, 60.60)	58.93 (57.84, 60.02)	58.45 (57.23, 59.67)	57.56 (56.55, 58.57)	0.284
Sex, %						0.181
Male	56.69 (54.12, 59.22)	51.80 (45.97, 57.59)	56.14 (51.62, 60.57)	58.18 (53.82, 62.42)	58.86 (54.20, 63.36)	
Female	43.31 (40.78, 45.88)	48.20 (42.41, 54.03)	43.86 (39.43, 48.38)	41.82 (37.58, 46.18)	41.14 (36.64, 45.80)	
Ethnicity, %						<0.001
Mexican American	9.08 (7.27, 11.28)	6.68 (4.79, 9.24)	10.18 (7.75, 13.28)	8.91 (6.88, 11.45)	9.93 (7.65, 12.79)	
Other Hispanic	5.25 (4.23, 6.51)	5.58 (4.04, 7.66)	6.37 (4.69, 8.61)	5.25 (4.09, 6.72)	4.23 (3.18, 5.60)	
Non-Hispanic White	65.44 (62.00, 68.73)	60.66 (54.97, 66.07)	62.39 (56.85, 67.63)	66.20 (62.11, 70.06)	70.02 (65.38, 74.29)	
Non-Hispanic Black	13.39 (11.39, 15.68)	20.29 (16.73, 24.39)	15.04 (12.01, 18.67)	12.32 (9.98, 15.12)	8.77 (7.07, 10.83)	
Other Ethnicities	6.84 (5.72, 8.16)	6.79 (4.64, 9.83)	6.01 (4.49, 8.02)	7.33 (5.61, 9.52)	7.05 (5.04, 9.77)	
Educational level, %						<0.001
< High school diploma	20.02 (18.30, 21.86)	32.62 (28.65, 36.85)	22.15 (18.91, 25.76)	19.38 (16.66, 22.41)	11.09 (9.12, 13.43)	
High school diploma	24.31 (22.42, 26.30)	25.21 (21.58, 29.22)	27.86 (24.00, 32.08)	21.71 (18.07, 25.85)	23.44 (20.14, 27.09)	
≥Some college	55.67 (53.40, 57.92)	42.17 (37.60, 46.88)	49.99 (45.43, 54.56)	58.92 (54.76, 62.95)	65.47 (61.19, 69.51)	
PIR	2.89 (2.80, 2.97)	2.28 ± 1.52	2.69 ± 1.60	2.99 ± 1.60	3.33 ± 1.55	<0.001
Hypertension, %						0.020
Yes	69.19 (66.88, 71.42)	72.38 (67.39, 76.87)	72.73 (68.73, 76.40)	68.72 (64.98, 72.23)	65.03 (60.52, 69.27)	
No	30.81 (28.58, 33.12)	27.62 (23.13, 32.61)	27.27 (23.60, 31.27)	31.28 (27.77, 35.02)	34.97 (30.73, 39.48)	
Hyperlipidemia, %						0.995
Yes	75.63 (73.70, 77.46)	75.97 (72.55, 79.08)	75.88 (70.96, 80.20)	75.28 (71.82, 78.44)	75.54 (71.24, 79.38)	
No	24.37 (22.54, 26.30)	24.03 (20.92, 27.45)	24.12 (19.80, 29.04)	24.72 (21.56, 28.18)	24.46 (20.62, 28.76)	
Diabetic kidney disease, %						<0.001
Yes	30.87 (28.98, 32.82)	42.75 (38.44, 47.16)	33.70 (29.82, 37.80)	28.66 (25.18, 32.42)	23.25 (20.19, 26.61)	
No	69.13 (67.18, 71.02)	57.25 (52.84, 61.56)	66.30 (62.20, 70.18)	71.34 (67.58, 74.82)	76.75 (73.39, 79.81)	

SD, standard deviation; PIR, family income-to-poverty ratio. For categorical variables: survey-weighted percentage (95% CI). For continuous variables: survey-weighted mean (95% CI).

### 3.2 Relationship between OBS and the risk of DKD in patients with T2DM

Table 3 shows the relationship between OBS and the risk of DKD in patients with T2DM using three multivariate linear regression models. The VIF for all covariates was below 10, indicating that there was no significant multicollinearity (refer to Supplementary Table S5) (43). The primary aim of the present investigation was to characterize the relationship between OBS and the risk of DKD in patients with T2DM using OBS as both a continuous and a categorical variable. After accounting for the impact of missing data, the findings remain consistent, as illustrated in the Supplementary Table S1. The findings indicate that a high OBS is associated with a reduced risk of DKD in Model 3 (odds ratio (OR), 0.97; 95% confidence interval (CI) 0.96–0.98). Specifically, for each unit increase in OBS, the likelihood of DKD in patients with T2DM decreases by 3%. When OBS was used as a categorical variable, the relationship remained significant. The risk of DKD decreased with increasing quartile in all the models. In Model 3, individuals with the highest OBS quartile had a 50% lower risk of DKD than those with the lowest OBS (OR 0.50; 95% CI 0.39–0.65).

**Table 3:** Results of the weighted logistic regression analysis of the relationship between OBS and the risk of DKD.

	Number of participants	Model 1 OR (95% CI)	Model 2 OR (95% CI)	Model 3 OR (95% CI)
OBS continuous	3,669	0.96 (0.94, 0.97)	0.96 (0.95, 0.97)	0.97 (0.96, 0.98)
Categories				
Q1	865	1.00 (ref)	1.00 (ref)	1.00 (ref)
Q2	904	0.68 (0.54, 0.85)	0.68 (0.54, 0.86)	0.71 (0.56, 0.90)
Q3	977	0.54 (0.41, 0.70)	0.55 (0.43, 0.71)	0.61 (0.47, 0.79)
Q4	923	0.41 (0.32, 0.52)	0.44 (0.34, 0.55)	0.50 (0.39, 0.65)
*p* for trend		<0.001	<0.001	<0.001
Dietary OBS (continuous)	3,669	0.96 (0.95, 0.98)	0.97 (0.95, 0.98)	0.97 (0.96, 0.99)
Category				
Q1	789	1.00 (ref)	1.00 (ref)	1.00 (ref)
Q2	937	0.64 (0.51, 0.81)	0.63 (0.49, 0.80)	0.65 (0.51, 0.84)
Q3	1,021	0.57 (0.43, 0.76)	0.59 (0.44, 0.77)	0.64 (0.49, 0.85)
Q4	922	0.43 (0.32, 0.57)	0.47 (0.36, 0.61)	0.54 (0.41, 0.71)
*p* for trend		<0.001	<0.001	<0.001
Lifestyle OBS (continuous)	3,669	0.87 (0.83, 0.92)	0.86 (0.81, 0.91)	0.89 (0.84, 0.95)
Category				
Q1	397	1.00 (ref)	1.00 (ref)	1.00 (ref)
Q2	1,307	0.77 (0.56, 1.07)	0.72 (0.52, 1.01)	0.72 (0.52, 1.00)
Q3	779	0.74 (0.52, 1.05)	0.67 (0.47, 0.96)	0.67 (0.47, 0.96)
Q4	1,192	0.50 (0.36, 0.69)	0.45 (0.32, 0.63)	0.45 (0.32, 0.63)
*p* for trend		<0.001	<0.001	0.002

Model 1 was unadjusted. Model 2 was adjusted for sex, year, and ethnicity. Model 3 was adjusted for sex, year, ethnicity, PIR, HLP, HBP, and educational level. OR, odds ratio; CI, confidence interval. OBS, oxidative balance score. Education level has six missing values (0.15%), and PIR has 336 missing values (8.40%).

Similarly, in patients with T2DM, we evaluated the protective effects of lifestyle and OBS using weighted logistic regression models. Individuals with T2DM in the second (OR 0.65, 95% CI 0.51–0.84), third (OR 0.64, 95% CI 0.49–0.85), and uppermost (OR 0.54, 95% CI 0.41–0.71) quartiles of dietary OBS had 35, 36, and 46% reduced risks of DKD, respectively, and dietary OBS was significantly associated with a lower risk of the presence of DKD. A similar relationship was found to exist between lifestyle OBS and DKD of patients with T2DM. In order to evaluate the potential influence of outliers on the outcomes of our analysis, we constructed a scatter plot of OBS versus DKD. No significant outliers were identified (refer to Supplementary Figure S1). The results are robust.

### 3.3 Results of the stratified analysis

We performed subgroup analyses to determine whether the association between OBS and the risk of DKD in patients with T2DM was consistent across participant categories. The *P* for the interaction was >0.05 for age, sex, ethnicity, educational level, and hypertension. However, hyperlipidemia had a significant effect on the association between OBS and DKD (*P* for interaction <0.05; Table 4). In T2DM with hyperlipidemia, OBS was associated with a lower risk of DKD (OR 0.96, 95% CI 0.95, 0.97), for patients without hyperlipidemia, although this trend was also observed, no statistical significance was found for the association (OR 0.98, 95% CI 0.96, 1.00).

**Table 4:** Results of the subgroup analysis of the relationship between OBS and the risk of DKD.

Characteristic	Number of participants	OR (95% CI)	*p* for interaction
Sex			0.4242
Male	2,136	0.96 (0.95, 0.97)	
Female	1,533	0.97 (0.96, 0.98)	
Age, years			0.2213
<40	294	1.00 (0.97, 1.04)	
41–60	1,241	0.98 (0.96, 0.99)	
≥60	2,134	0.96 (0.95, 0.97)	
Ethnicity			0.4462
Mexican American	627	0.96 (0.94, 0.99)	
Other Hispanic	390	0.98 (0.95, 1.01)	
Non-Hispanic White	1,404	0.97 (0.95, 0.98)	
Non-Hispanic Black	927	0.96 (0.94, 0.98)	
Other Ethnicities	321	0.95 (0.92, 0.98)	
Hypertension			0.4844
Yes	2,606	0.96 (0.95, 0.97)	
No	1,063	0.98 (0.96, 1.00)	
Hyperlipidemia			0.0382
Yes	2,712	0.96 (0.95, 0.97)	
No	957	0.98 (0.96, 1.00)	
Education			0.277
< High school diploma	1,104	0.96 (0.94, 0.97)	
High school diploma	857	0.98 (0.96, 1.00)	
≥Some college	1,708	0.96 (0.95, 0.98)	

OR, odds ratio; CI, confidence interval.

**Figure 2:**
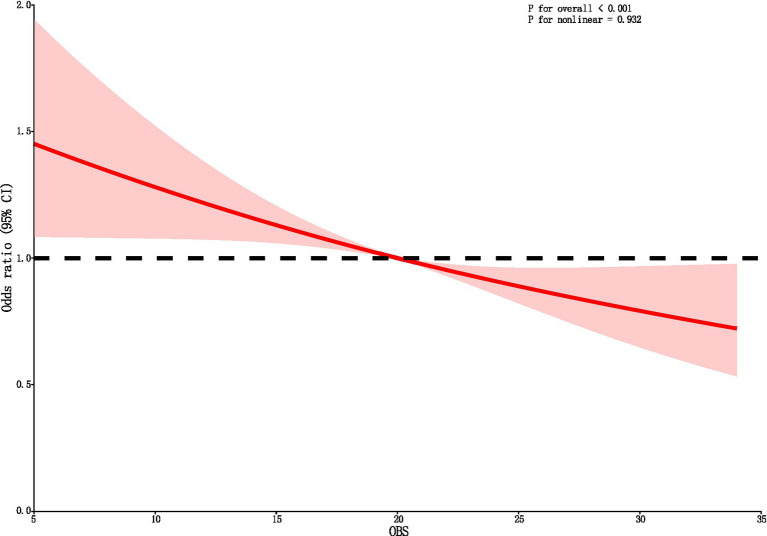
The RCS curve of the association between OBS and DKD. RCS regression was adjusted for sex, year, ethnicity, PIR, HLP, HBP, and educational level. OBS, oxidative balance score; RCS, restricted cubic spline; CI, confidence interval.

Following the adjustment for all covariates, the RCS curve demonstrates a linear negative correlation between OBS and DKD (Figure 2).

## 4 Discussion

In the present study, we conducted a cross-sectional analysis of data from the 2017–2018 NHANES to characterize the association between OBS and the risk of DKD among individuals with T2DM. As far as we are aware, this is the first such investigation of this risk in people with T2DM. After controlling for potential confounding variables and separately evaluating the independent relationships of lifestyle OBS and dietary OBS with DKD, we obtained the same results: that a high OBS score is inversely associated with the risk of DKD in patients with T2DM. This finding emphasizes the importance of the consumption of a diet rich in antioxidants and the adoption of healthy habits by individuals with diabetes to reduce the risk of developing renal complications. Consequently, by evaluating the dietary habits and lifestyle factors of patients with T2DM and calculating their OBS, healthcare professionals can gain insight into their risk of developing DKD. To provide a foundation for the personalized management strategy for patients with T2DM.

Overall, the present findings are similar to those of previous studies. These studies (31, 32) were most frequently conducted in the general population and were cross-sectional in nature. An association between CKD risk and OBS quartile was found by Wu et al. (29). However, they did not identify a significant effect of dietary OBS on the risk of CKD. In addition, there have also been studies of the relationship between specific components of OBS and DKD in patients with diabetes (44). Through interaction testing, we found that hyperlipidemia is a confounder of the association between OBS and DKD. Hyperglycemia and hyperlipidemia cause OS, which results in an increase in the circulating concentration of advanced glycation end-products through multiple mechanisms and can speed up DKD progression (45, 46). This suggests that T2DM patients with hyperlipidemia should strive to maintain a higher OBS to decrease the risk of DKD. Furthermore, hyperlipidaemia exacerbates oxidative stress via its effect on lipid peroxidation and ROS production. Excess ROS can directly damage renal cells and result in inflammation and fibrosis. Hyperlipidaemia also impacts mitochondrial function and causes endoplasmic reticulum stress, further contributing to oxidative stress. Therefore, further investigation is required to elucidate the precise mechanism via which hyperlipidaemia influences the risk of DKD.

Hyperglycemia is the principal cause of DKD (47). High glucose concentration damages mitochondria, which increases the production of ROS (48), and OS develops when the generation of ROS surpasses the innate capacity of cells to clear antioxidants, leading to significant local tissue damage (9, 11).

OS is regarded as a key cause of complications related to T2DM, and especially renal complications (49). The impact of most components of the OBS on DKD was previously discussed. The ingestion of fiber affects the composition of the intestinal microbiota, resulting in greater microbial fermentation and the production of short-chain fatty acids, which reduce the inflammation and fibrosis associated with DKD (50, 51). This mechanism may provide a new perspective for reducing the risk and managing DKD.

Vitamin B is principally obtained through the diet and has antioxidant effects, thereby protecting against OS (52). Alam et al. found that the OS of diabetic mice is significantly ameliorated by Vitamin B2 (riboflavin) supplementation, leading to a reduction in renal tissue damage (53). Another study showed that Vitamin B3 (niacin) supplementation reduces the serum phosphate concentrations of patients with renal failure who are undergoing hemodialysis, including those with DKD (54). The deficiency of Vitamin B6 (pyridoxine) in patients with diabetes predisposes toward DKD through various mechanisms, such as an increase in ROS formation and microvascular damage (52, 55). In addition, cobalamin insufficiency may lead to decreased antioxidant enzyme activity, which increases oxidative stress and exacerbates the progression of DKD (52, 56). Chan et al. also reported that folic acid deficiency may worsen the inflammation associated with CKD and exacerbate renal fibrosis (57). Omar et al. discovered that vitamin C has a protective effect against lipid peroxidation, which may reduce OS and inflammation in patients with CKD who are undergoing hemodialysis (58). Vitamin E, and especially its tocotrienol isomers, has potent antioxidant and anti-inflammatory properties (59), and dietary supplementation with tocotrienol-rich vitamin E for 12 months has been shown to slow the progression of DKD (60).

Alcohol consumption, smoking, and obesity are known to cause increases in OS and mitochondrial damage. In addition, the importance of physical activity in patients with DKD has also been studied (61). Each of these modifiable factors associated with lifestyle (physical activity, diet, alcohol consumption, and smoking) individually affect REDOX homeostasis in the body, thereby having an effect on the progression of DKD (62, 63). Thus, lifestyle interventions that help manage the risk CKD, including DKD, include increasing physical activity, reducing alcohol consumption, and quitting smoking (64). To establish a causal relationship between OBS and DKD, future research should prioritize large-scale longitudinal studies that monitor changes in OBS over time and examine their association with the incidence and progression of DKD. Furthermore, it will be crucial to examine the influence of interventions designed to increase OBS, such as dietary modifications that boost antioxidant intake and lifestyle modifications that minimize exposure to pro-oxidants, on kidney disease outcomes. The evaluation of these strategies may provide valuable insights for the prevention and management of DKD.

In conclusion, maintaining oxidative balance is paramount for managing the risk of DKD. OS is regarded as a key cause of T2DM-associated complications, and especially renal complications (49). The impacts of most components of the OBS on DKD were previously discussed. The ingestion of fiber affects intestinal microbiota composition, resulting in increased microbial fermentation and the production of short-chain fatty acids, which reduce the inflammation and fibrosis associated with DKD (50, 51). This may provide a new perspective for reducing the risk and managing DKD.

However, the mechanisms by which individual OS-related defects affect the progression of DKD is not fully understood. For example, the specific relationship between vitamin B deficiency and OS in patients with DKD has not been characterized. Copper and zinc homeostasis is tightly regulated, and an imbalance in these minerals can result in insulin resistance and OS. Nevertheless, the mechanisms underlying the protective or harmful effects of copper and zinc in diabetes and DKD are complex (65).We believe that it is more important to focus on OBS than its individual elements. Therefore, we used OBS to comprehensively assess the pro-oxidant and antioxidant exposure status of the participants and to investigate the overall effect on the risk of DKD in patients with T2DM.

In addition to those already mentioned above, the present study has several other strengths. First, the sample size was large and representative of a wide population, and second, we adjusted the data for potential confounding covariates to improve the robustness of the findings. However, the study also has some limitations. First, the effects of unknown or unmeasured confounders cannot be eliminated. Second, the potential impact of interventions targeting OBS on the risk of DKD was not explored, and we did not have access to long-term follow-up data. Finally, the study design was cross-sectional, which limited its ability to infer causal relationships.

## 5 Conclusion

In summary, the present cross-sectional study has revealed negative associations of lifestyle OBS and dietary OBS with the risk of DKD in patients with diabetes. Nevertheless, further prospective studies are warranted to validate the findings.

## Data Availability

The original contributions presented in the study are included in the article/Supplementary material, further inquiries can be directed to the corresponding author/s.

## Footnotes

^1^www.empowerstats.net, X&Y Solutions, Inc.
