# Supplementary material for: Oxidative balance score is associated with the risk of diabetic kidney disease in patients with type 2 diabetes mellitus: evidence from NHANES 2007–2018
Source: Front Nutr. 2024 Dec 19;11:1499044. doi: 10.3389/fnut.2024.1499044 (PMC11693591; doi:10.3389/fnut.2024.1499044)
Supplement: Supplementary file 1 [file Table_1.docx]

**Supplementary Table 1. Results of the Weighted Logistic Regression Analysis of the Relationship Between OBS and the Risk of DKD After Missing Value Imputation**

|  | Number of participants | Model 1 OR (95% CI) | Model 2 OR (95% CI) | Model 3 OR (95% CI) |
| --- | --- | --- | --- | --- |
| OBS continuous | 4011 | 0.96 (0.95, 0.98) | 0.96 (0.95, 0.97) | 0.97 (0.96, 0.98) |
| Categories |  |  |  |  |
| Q1 | 859 | 1.00 (ref) | 1.00 (ref) | 1.00 (ref) |
| Q2 | 991 | 0.83 (0.65, 1.07) | 0.79 (0.60, 1.03) | 0.78 (0.60, 1.04) |
| Q3 | 1108 | 0.67 (0.52, 0.86) | 0.63 (0.50, 0.81) | 0.67 (0.53, 0.86) |
| Q4 | 1053 | 0.47 (0.37, 0.61) | 0.46 (0.36, 0.59) | 0.51 (0.39, 0.65) |
| *P* for trend |  | <0.001 | <0.001 | <0.001 |
| Dietary OBS (continuous) | 4011 | 0.96 (0.95, 0.98) | 0.97 (0.95, 0.98) | 0.97 (0.96, 0.98) |
| Category |  |  |  |  |
| Q1 | 861 | 1.00 (ref) | 1.00 (ref) | 1.00 (ref) |
| Q2 | 1023 | 0.75 (0.59, 0.96) | 0.73 (0.56, 0.94) | 0.74 (0.57, 0.96) |
| Q3 | 1117 | 0.61 (0.48, 0.78) | 0.59 (0.47, 0.76) | 0.63 (0.50, 0.80) |
| Q4 | 1010 | 0.47 (0.36, 0.62) | 0.50 (0.39, 0.65) | 0.55 (0.42, 0.71) |
| *P* for trend |  | <0.001 | <0.001 | <0.001 |
| Lifestyle OBS (continuous) | 4011 | 0.93 (0.87, 0.99) | 0.85 (0.80, 0.91) | 0.87 (0.81, 0.93) |
| Category |  |  |  |  |
| Q1 | 648 | 1.00 (ref) | 1.00 (ref) | 1.00 (ref) |
| Q2 | 758 | 0.87 (0.65, 1.16) | 0.74 (0.54, 1.01) | 0.75 (0.54, 1.02) |
| Q3 | 950 | 0.77 (0.57, 1.03) | 0.59 (0.44, 0.80) | 0.61 (0.45, 0.82) |
| Q4 | 1655 | 0.73 (0.55, 0.98) | 0.52 (0.38, 0.71) | 0.56 (0.41, 0.76) |
| P for trend |  | 0.032 | <0.001 | <0.001 |

Model 1 was unadjusted.

Model 2 was adjusted for sex, year, and ethnicity.

Model 3 was adjusted for sex, year, ethnicity, PIR, HLP, HBP, and educational level.

OR, odds ratio; CI, confidence interval. OBS, oxidative balance score

**Supplementary Table 2. Relationship between covariates and DKD**

| Covariates | N | term | beta | Se. | 95%CI Low | 95%CI Upp | P.value |
| --- | --- | --- | --- | --- | --- | --- | --- |
| Sex | 3669 | factor(SEX)2 | -0.0886 | 0.0697 | 0.7984 | 1.0491 | 0.2033 |
| Year | 3669 | YEAR | 0.0375 | 0.0029 | 1.0324 | 1.0441 | <0.0001 |
| Race | 3669 | factor(RACE)2 | -0.0465 | 0.1375 | 0.7291 | 1.2498 | 0.7351 |
|  |  | factor(RACE)3 | 0.1133 | 0.1014 | 0.9182 | 1.3661 | 0.2635 |
|  |  | factor(RACE)4 | 0.4481 | 0.1075 | 1.268 | 1.9325 | <0.0001 |
|  |  | factor(RACE)5 | -0.1735 | 0.1488 | 0.6281 | 1.1253 | 0.2434 |
| PIR | 3669 | PIR | -0.151 | 0.0227 | 0.8224 | 0.899 | <0.0001 |
| HLP | 3669 | factor(HLP)1 | 0.3107 | 0.0801 | 1.1661 | 1.5962 | 0.0001 |
| HBP | 3669 | factor(HBP)1 | 0.8306 | 0.0822 | 1.953 | 2.6961 | <0.0001 |
| Education level | 3669 | factor(EDUCATION)2 | -0.1125 | 0.0935 | 0.7439 | 1.0734 | 0.2291 |
|  |  | factor(EDUCATION)3 | -0.2728 | 0.0799 | 0.6508 | 0.8903 | 0.0006 |

**Supplementary Table 3. Impact of Covariates on OBS Regression Coefficient**

|  |  | Basic model | Complete model |  |
| --- | --- | --- | --- | --- |
| Covariates | +/- term | OBS | OBS | elect |
|  | Initial regression coefficient | -0.0378 | -0.0242 |  |
| Sex | Factor (SEX) | -0.038 | -0.0242 |  |
| Year | YEAR | -0.0338 * | -0.0271 * | Yes |
| Race | Factor (RACE) | -0.0351 | -0.0272 * | Yes |
| PIR | PIR | -0.0325 * | -0.0289 * | Yes |
| HLP | Factor (HLP) | -0.0378 | -0.0242 |  |
| HBP | Factor (HBP) | -0.0354 | -0.0249 |  |
| Education level | Factor (EDUCATION) | -0.0359 | -0.0233 |  |

* Indicates a change of more than 10% from the initial regression coefficient.

**Supplementary Table 4. Filtered covariates**

| Y | X | Selected covariates  (Criterion 1) | Selected covariates  (Criterion 2) |
| --- | --- | --- | --- |
| DN | OBS | Year Race PIR | Year Race PIR HLP HBP  Education level |

Notes:

1. Criterion 1: The effect of introducing covariates in the basic model or removing covariates from the full model on the regression coefficient of OBS is >10%.

2. Criterion 2: The regression coefficient of criterion 1 or covariate on DKD has a P value <0.1.

**Supplementary Table 5.VIF collinear screening**

|  | VIF |
| --- | --- |
| Sex | 1 |
| Year | 1.1 |
| Race | 1.1 |
| PIR | 1.2 |
| HLP | 1 |
| HBP | 1.1 |
| Education level | 1.3 |


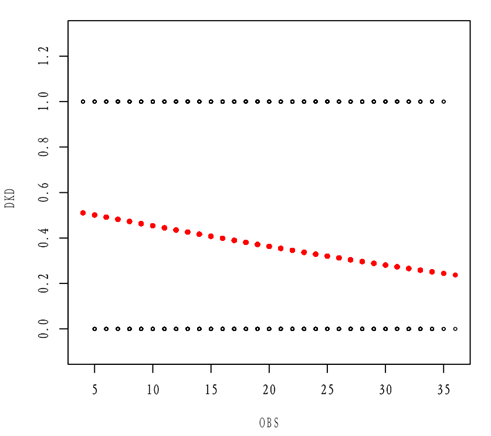


**Supplementary Figure. Scatter diagram**

X axis: Indicates the Oxidation Balance Score (OBS)；Y axis: Indicates Diabetic kidney disease(DKD)；Each point representing a study participant
